# Low Back Pain and Associated Factors among Primary School Teachers in Mekele City, North Ethiopia: A Cross-Sectional Study

**DOI:** 10.1155/2019/3862946

**Published:** 2019-07-08

**Authors:** Aregawi Kebede, Solomon Mekonnen Abebe, Haile Woldie, Melaku Kindie Yenit

**Affiliations:** ^1^Mekele City Health Office, Mekele, Ethiopia; ^2^Department of Human Nutrition, College of Medicine and Health Science, Institute of Public Health, University of Gondar, Gondar, Ethiopia; ^3^Department of Epidemiology and Biostatistics, College of Medicine and Health Science, Institute of Public Health, University of Gondar, Gondar, Ethiopia

## Abstract

**Background:**

Low back pain (LBP) is the most prevalent musculoskeletal disorder among teachers. The pain, which is widely considered to be caused by occupational activities, has a significant impact on teachers' quality of life because it results in frequent sick leaves, functional impairment, and early retirement. It is also demanding in terms of treatment costs, individual suffering, and discontinuation of jobs. Therefore, this study assessed the magnitude of low back pain and associated factors among primary school teachers in Mekele City, north Ethiopia.

**Methods:**

An institution-based cross-sectional study which included 611 public primary school teachers of Mekele City was conducted from March to April 2015. A multivariable logistic regression analysis was used to identify factors associated with low back pain. The adjusted odds ratio (AOR) with a 95% confidence interval (CI) was used to show the strength of the associations, and variables with a *p* value of less than 0.05 were considered statistically significant.

**Results:**

In this study, the prevalence of low back pain was 74.8% (95% CI: 71.4-78.2). According to the multivariate analysis, the pain was associated with teachers' sleeping disturbance (AOR = 6.99; 95% CI: 2.20, 13.63), prolonged standing (AOR = 4.78; 95% CI: 3.75, 9.32), and irregular physical exercise (AOR = 1.46; 95% CI: 1.29, 5.10).

**Conclusion:**

The study showed that the prevalence of low back pain was high. Sleeping disturbance, prolonged standing during sessions, and irregular physical activity were significantly associated with the problem. Therefore, addressing work-related and individual factors is essential for decreasing the burden.

## 1. Background

Musculoskeletal disorders are major health problems that cause disability and result in a considerable impact on the quality of life leading to economic burden in terms of compensation costs, lost wages, and unproductivity. It is the major cause of pain that most people complain about and is a warning signal for the risk of tissue damage. Globally, it is the second cause of disability and the fourth greatest impact on health. Around the world, hundreds of millions of people are severely affected by musculoskeletal disorders that cause long-term pain and physical disability with serious effects on workers' health and productivity [1, 2]. The World Health Organization (WHO) defined musculoskeletal disorders as “a disorder of muscles, tendons, joints, intervertebral discs, peripheral nerves, and vascular systems, not directly resulting from an acute or instantaneous event but installing gradually and chronically” [3–5].

Low back pain (LBP), also known as “*lumbago*”, represents one of the most common musculoskeletal disorders which are common problems among the working population, including teachers [1, 6, 7]. It has a substantial effect on school teachers' quality of life, resulting in frequent sick leaves, functional impairment, absenteeism, and early retirement. The tasks of school teachers might be subjected to conditions that involve a constant use of a “Head Down” posture during frequent readings, marking assignments, and writing on boards. Poor posture and improper techniques of lifting or carrying are the two very common causes of low back pain. Lifting heavy loads and materials, such as books, overhead projectors, and other equipment rank as the main contributing factors [8–10]. In addition, the work of teachers is not only limited to teaching students but also includes preparing lessons, assessing students' work, and being involved in extracurricular activities such as sports [11]. These variations may expose teachers to adverse mental and physical health effects [9].

LBP is the most prevalent musculoskeletal condition, commonly causing disability in both developed and developing countries. It is estimated that about 70% to 85% of the global population experience low back pain at some time in their life [4]. In developed countries, the problem ranges from 26.4% to 79.2% [12–17]. Especially among teachers, its prevalence is much higher compared to that of other occupational groups, ranging from 12% to 95% [16, 18–21]. Literature indicates that low back pain is caused by various factors mainly associated with occupational and individual factors. Work-related tasks, such as prolonged sitting, awkward posture, and other psychological and organizational factors, are the major causes of LBP, especially among teachers.

Though musculoskeletal disorders are a common problem among the working population, specifically within the teaching profession, it has not been given sufficient attention in the literature. Teaching by itself represents a high risk for musculoskeletal disorders, and prolonged exposure to unfavorable working conditions during teaching becomes a health risk [10]. Therefore, this study assessed the prevalence and associated factors of low back pain among primary school teachers in Mekele City, north Ethiopia [22].

## 2. Methods

### 2.1. Study Design and Setting

An institution-based cross-sectional study was conducted from March to June 2015, in Mekele City, north Ethiopia. The study was conducted on public primary school teachers in the city, located 780 km north of the capital of Ethiopia, Addis Ababa. According to the 2013 report of the Central Statistical Agency of Ethiopia, the city had a total population of more than 286,600 people [23]. In the city, there were 26 high schools, 15 first-cycle primary schools, and 64 second-cycle primary schools. Out of the 2856 first- and second-cycle teachers in the public and private schools, 48.7% were female.

### 2.2. Study Participants, Sample Size, and Sampling Procedure

Teachers of the selected primary schools, except those on sick and other leaves, participated in the study. The sample size was calculated using Epi Info version 3.7 by considering a 53.8% prevalence of low back pain [21], a 95% level of confidence, a 4% margin of error, and a 10% nonresponse rate. Finally, a sample of 656 was obtained. Then, teachers working in public primary schools were proportionally assigned to each school and selected by using computer-generated random numbers.

### 2.3. Data Collection Procedure

A structured self-administered questionnaire adopted from the Nordic musculoskeletal tool (Standardized Nordic Questionnaire) was used to collect data [24]. A questionnaire validated by Scandinavian countries and customized to our setting was used as an instrument for collecting neck and back musculoskeletal symptoms. To maintain consistency, the questionnaire was first translated from English to Tigrigna (the native language of the study area) and retranslated to English by professional translators and physiotherapists. Three nurse data collectors and a senior physiotherapist supervisor were selected. A two-day training on the objectives of the study and about the confidentiality of information was given to data collectors and the supervisor. The questionnaire was also piloted on 5% of the total sample from the study area to evaluate the acceptability and applicability of the procedures. Moreover, the reliability of the questionnaire was tested and a reliable Cronbach's alpha score was found (Cronbach′s alpha = 0.82).

### 2.4. Operational Definitions and Study Variables

Low back pain is defined as a perceived self-reported pain and/or discomfort, localized between the coastal margin (bottom of the ribs) and above the inferior gluteal folds (top of the legs) which has lasted for days or weeks during the last 12 months [3]. Acute and subacute low back pain is also defined as pain that lasts less than 6 weeks and 6-12 weeks, respectively, while chronic low back pain is defined as a pain that lasts 12 weeks and longer.

### 2.5. Data Processing and Analysis

Data were entered using Epi Info version 3.5.3 and exported to the Statistical Package for Social Sciences (SPSS) version 20 for further analysis. For missing data, the author omitted such cases and analyzed what remained—complete case analysis. Descriptive statistics, like frequencies and proportions, were computed, and the binary logistic regression model was used to identify significant variables. Variables with a *p* value of less than 0.2 in the bivariable logistic regression analysis were entered into the multivariable logistic regression analysis. Both crude odds ratio (COR) and adjusted odds ratio (AOR) with the corresponding 95% confidence intervals were calculated to show the strength of the associations. To control the possible effects of confounders, multivariable logistic regression analysis was done. Finally, a *p* value of less than 0.05 at the multivariable logistic regression analysis was used to identify variables significantly associated with neck and/or back pain.

## 3. Result

### 3.1. Sociodemographic and Economic Characteristics

A total of 611 public primary school teachers were included with a response rate of 93%. Over half (54.2%) of the respondents were females; the mean (standard deviation) age of the teachers was 40 (±9.38) years. One-quarter (25.4%) of the respondents were overweight (BMI: 25-29.9 kg/m^2^). A majority (93.8% and 79.9%, respectively) of the respondents were Orthodox Christians and diploma graduates. Nearly two-thirds (62.4%) were married, and a bit more than half (55.3%) served for 11-24 years. Only 6.4% of the teachers had an extra source of income (Table 1).

### 3.2. Work-Related Characteristics of Respondents

More than three-quarters (76.5%) of the teachers worked in a prolonged standing position. The mean (standard deviation) time of standing per day without any break was 3.6 (±1.6) hours. The majority (90.7%) delivered lectures walking from class to class, and nearly half (41.9%) had less than 20 minutes of break between sessions. Almost one-third (32.4% and 28%, respectively) of the school teachers had over 30 hours of teaching per week and less than 6 hours of sleep per day. About 37.5% of the teachers reported that they had sleeping disturbances.

One-quarter (25.6%) of the teachers were exposed to prolonged sitting due to curricular activities, and the mean (standard deviation) time per day for sitting was 2.8 (±2.3) hours. The most reported reason for sitting for a long time in teaching was marking exams and assignments. More than half (56.0% and 59.5%, respectively) of the teachers reported that they usually used bench chairs for sitting and had one-per-week shift patterns. Only 10.3% of the teachers had separate offices. The majority (96.9%) used teaching aids and devices, while one-third (33.7%) of them lifted heavy loads. About 78.3% of the teachers had off-school jobs. Only a bit higher than one-third (38.0%) took training on work place safety issues. It was reported that about 45.3% of the teachers were satisfied with their job, and higher than half (53.2%) had discouragement and depression during work, while one-third (33.1%) reported that they faced verbal or physical violence at work. Two-thirds (64.0%) of the teachers said they were irritated or angry with either their family or supervisors or immediate bosses (Table 2).

### 3.3. Comorbidity and Psychosocial Characteristics

One-third (32.4% and 33.7%, respectively) of the teachers had a history of low back pain (LBP) and recurrent severe headaches. A significant proportion (60.9%) of the respondents reported that they had stresses related to income, health, work, or family. Nearly one-fifth (16.5%) of the teachers said that they had asthma, and 69.1% felt low back pain during their fits of coughing (Table 3).

### 3.4. Prevalence of Low Back Pain

In this study, the overall prevalence of low back pain in the last 12 months was 74.8% (95% CI: 71.4%-78.2%). The prevalence was substantially higher among women (82.2%) than among men (66.1%). Low back pain among 1st- and 2nd-cycle school teachers was almost comparable at 79.9% and 70.7%, respectively. One-third of the teachers reported a history of low back pain. It was stated that prolonged standing was the most probable cause of low back pain among teachers, and the majority (93.2%) developed the pain after their employment. The duration of low back pain extended up to 12 weeks for about one-third (30.1%) of the teachers.

### 3.5. Factors Associated with Low Back Pain

In bivariable logistic regression analysis, sex, age, marital status, work experience, monthly income, sleeping disturbance, stress, irregular physical exercise, prolonged standing, length of break, asthma, and previous back injury were found to be factors associated with low back pain at a *p* value of less than 0.2. Consequently, after these variables were subjected to multivariate logistic regression analysis, prolonged sleeping disturbance and irregular physical exercise were found statistically significant and independently associated with low back pain at a *p* value of less than 0.05.

Accordingly, higher odds of low back pain were noted among teachers who had sleeping disturbances compared to those who had no such problems (AOR = 6.99; 95% CI: 2.20, 13.63). The odds of low back pain among teachers who had a sleeping disorder were almost seven times higher compared with teachers who had normal sleep. Likewise, the odds of low back pain were higher among teachers who had no regular physical exercises (AOR = 1.46; 95% CI: 1.29, 5.10) compared with those who had adequate exercises. It was noted that the odds of low back pain among teachers who had regular physical exercise were 1.46 times higher than among teachers who had no regular physical exercises. Moreover, the odds of low back pain were higher among teachers who had prolonged standing during sessions (AOR = 4.78; 95% CI: 3.75, 9.32) compared with their counterparts. It was also reported that low back pain among respondents with prolonged standing at sessions was almost five times higher than among teachers with no prolonged standing (Table 4).

## 4. Discussion

In this study, the overall prevalence of low back pain in the last 12 months was 74.8% (95% CI: 71.4%-78.2%). The result was in line with those of studies conducted in Iran (71.9%) and Turkey (74.9%) [16]. However, it was higher than those of studies conducted in Saudi Arabia (63.8%), the Philippines (53.3%), Brazil (41.1%), India (40.4%), Botswana (55.7%) [9], China (59.2%), Malaysia (40.4%) [25], and Japan (20.6%). The prevalence of low back pain was higher in this study compared to what was reported by these previously mentioned countries, perhaps because of poor facilities in our schools, low morale, and the poor income of teachers owing to socioeconomic differences among the settings in which the studies were done. In addition, low awareness about how to decrease and control such work place hazards, the way work is organized, and the excessive work load of primary school teachers could be considered as factors contributing to the differences observed in the studies in the other places. Moreover, the high proportion of female respondents might be the other justification for the greater prevalence in our study. This is because women teachers are culturally and socially obliged to perform almost all home activities which results in a heavier work load and greater risk to LBP than male teachers. In addition, since women tend to have a lower pain threshold than men, they are more likely to report problems than men.

The result of the adjusted analysis indicated that sleeping disturbance, regular physical exercise, and prolonged standing were significantly associated with LBP. It was revealed that the odds of LBP were higher among teachers who had sleeping disturbances. Teachers who felt discomfort or were disturbed during sleep were almost seven times more likely to experience low back pain compared with teachers who felt comfortable or were not disturbed during sleep (AOR=6.99; 95% CI: 2.20, 13.63). This was in agreement with the reports from other African and Asian countries [15]. The possible reason for this might be the fact that teachers who lack sufficient sleep would not get as much rest as teachers who get sufficient sleep. In other words, this might be related to the stress that could be associated with unhealthy sleepless nights which predispose teachers to low back pain; this may be due to prolonged sitting when sleepless. Reports also show that teachers who have sleeping disturbances may feel constantly under strain and unhappy or stressed and face a significant psychological risk factor for musculoskeletal disorders.

In line with other findings elsewhere [10, 17–19], the odds of low back pain in this study were positively associated with the length of standing during sessions. It is specifically high among teachers who have longer standing periods in sessions. The odds of LBP among teachers with prolonged standing were almost five times higher than among teachers with breaks between sessions (AOR = 4.78; 95% CI: 3.75, 9.32). This may be due to the effect of physical efforts during teaching, including prolonged standing in an inappropriate way for several hours inside the classroom resulting in an excessive strain on the lumbar spine, subsequently leading to back and musculoskeletal pain among teachers. Most teachers experience the same standing position risk for the development of lower back pain. Standing long might not be the only factor for low back pain; activities such as carrying loads and teaching resources to schools or classrooms, and walking in and outside the school may aggravate the pain. In addition, twisting, such as turning from the board to the class and back again, prolonged sitting to mark exams and prepare teaching documents, unsuitable furniture, and lifting loads are also risk factors for low back pain.

Furthermore, the odds of low back pain among physically inactive teachers were high compared with physically active teachers (AOR = 1.46: 95% CI 1.29, 5.10). The finding is similar to reports from Athens. This could be due to the fact that weak and shortened muscles can cause a misalignment of the spine, while regular physical exercises can strengthen the muscles to support and maintain the spine in perfect alignment for proper functioning [26].

Though the study did its best to indicate the magnitude of low back pain among primary school teachers, it is not free from limitations. The cross-sectional design might have prevented the work from showing temporal relationships. In addition, since low back pain has not been verified by clinical diagnosis in the last 12 months, our result is based on self-reporting. Thus, it is possible that participants fail to remember correctly and end up in a recall bias. Although the Nordic musculoskeletal tool is a standardized questionnaire to measure low back pain and musculoskeletal disorder, it is not validated for the study setting.

## 5. Conclusion

In the study setting, the magnitude of low back pain among primary school teachers was high, and sleeping disturbance, prolonged standing during sessions, and physical inactivity were found statistically significant and independently associated with it. Thus, addressing work-related and individual factors are essential to decrease the burden of the problem.

## Figures and Tables

**Table 1:** Sociodemographic and economic characteristics of primary school teachers in Mekele City, north Ethiopia, 2017.

Variables	Frequency	Percent
Sex		
Male	280	45.8
Female	331	54.2
Age (in years)		
<30	139	22.7
31-40	212	34.6
Above 40	260	42.7
Body mass index		
Less than 18.5	39	6.2
18.5-24.9	291	47.6
25-29.9	155	25.4
≥30	126	20.8
Religion		
Orthodox	573	93.7
Muslim	26	4.3
Protestant	8	1.3
Catholic	4	0.7
Educational status		
Certificate	24	3.9
Diploma	488	79.9
Degree and above	99	16.2
Marital status		
Single	128	20.9
Married	381	62.4
Divorced/widowed	102	16.7
Work experience (in years)		
<10	119	19.5
11-24	338	55.3
≥25	154	25.2
Monthly salary (ETB)		
>3501	265	43.4
2501-3500	251	41.1
<2500	95	15.5
Have extra source of income		
No	572	93.6
Yes	39	6.4

**Table 2:** Work-related characteristics of primary school teachers in Mekele City, north Ethiopia, 2017.

Variables	Frequency	Percent
Prolonged standing during teaching		
Yes	467	76.5
No	144	23.5
Length of standing during teaching per session		
2-5 hours	295	63.0
More than 5 hours	172	37.0
Frequency of teaching class		
One class per week	57	9.3
Moving from class to class	554	90.7
Length of break time between sessions		
Less than 20 minutes	256	41.9
Up to 1 hour	228	37.3
More than 1 hour	103	16.8
Working hours per week		
<30 hours	413	67.6
≥30 hours	198	32.4
Sleeping time per day		
<6	171	28.0
6-9	342	56.0
≥10	98	16.0
Sleeping disturbance		
Yes	229	37.5
No	382	62.5
Have prolonged sitting		
Yes	156	25.6
No	454	74.4
Reasons of prolonged sitting at work		
For exam marking	87	55.8
For assignment marking	56	35.9
Other reasons	13	8.3
Type of seat frequently used at office		
Swivel chair	12	2.0
Normal chair	257	42.0
Bench	342	56.0
Work shift pattern		
Only morning	105	17.2
Whole shift	142	23.3
Once per week	363	59.5
Have separate office		
Yes	64	10.3
No	547	89.7
Shifting heavy materials		
Yes	206	33.7
No	405	66.3
Have extracurricular activity		
Yes	478	78.3
No	133	21.7
Have regular physical exercise		
Yes	423	69.2
No	188	30.8
Time for physical exercise per week (in hours)		
≤5	451	74.6
>5	156	25.4
Have training on safety at work		
Yes	232	37.9
No	379	62.1
Satisfaction with working environment		
Yes	277	45.3
No	334	54.7
Have discouragement and depression		
Yes	325	53.2
No	286	46.8
Experience of any verbal or physical violence		
Yes	202	33.1
No	409	66.9

**Table 3:** Comorbidity- and psychosocial-related characteristics of primary school teachers in Mekele City, north Ethiopia, 2017.

Variables	Frequency	Percent
History of LBP injury		
Yes	198	32.4
No	413	67.6
Have stress		
Yes	372	60.9
No	239	39.1
Recurrent severe headache		
Yes	206	33.7
No	405	66.3
Feeling LBP during headache		
Yes	152	73.8
No	54	26.2
Have asthma		
Yes	101	16.5
No	510	83.5
Feeling LBP during asthma		
Yes	70	69.1%
No	31	76.4

**Table 4:** Factors associated with low back pain among primary school teachers in Mekele City, north Ethiopia, 2017.

Variables	Low back pain		Crude odds ratio	Adjusted odds ratio
	Yes (%)	No (%)	(95% CI)	(95% CI)
Sex				
Male	185 (66.1)	95 (33.9)	1	1
Female	272 (82.2)	59 (17.8)	2.36 (1.62, 3.44)	3.23 (0.69, 15.02)
Age (years)				
<30	92 (66.2)	47 (33.8)	1	
30-40	156 (73.6)	56 (26.4)	1.42 (0.89, 2.26)	0.30 (0.72,1.33)
≥41	209 (80.4)	51 (19.6)	2.09 (1.31, 3.33)	0.42 (0.56, 2.42)
Marital status				
Single	79 (61.7)	49 (38.3)	1	
Married	307 (80.6)	74 (19.4)	2.02 (1.32, 3.09)	2.70 (0.80, 9.10)
Other^∗^	71 (69.6)	31 (30.4)	4.00 (1.93, 8.29)	4.07 (0.75, 22.15)
Work experience (in years)				
<10	59 (46.1)	69 (53.9)	1	1
11-24	253 (66.4)	128 (33.6)	2.13 (1.35, 3.34)	2.56 (0.38, 14.00)
>25	54 (52.9)	48 (47.1)	2.14 (1.26, 3.65)	2.64 (0.29, 19.29)
Monthly income (ETB)				
>3501	209 (78.9)	56 (21.1)	2.38 (1.43, 3.95)	1.15 (0.21, 6.38)
2501-3500	190 (75.7)	61 (24.3)	1.98 (1.20, 3.28)	1.01 (0.19, 5.43)
<2500	58 (61.1)	37 (38.9)	1	1
Sleeping disturbance				
Yes	196 (85.6)	33 (14.4)	2.75 (1.79, 4.22)	6.99 (2.20, 13.63)
No	261 (68.3)	121 (31.7)	1	1
Have stress				
Yes	302 (81.2)	70 (18.8)	2.33 (1.61, 3.39)	1.07 (0.48, 2.37)
No	155 (64.9)	84 (35.1)	1	1
Regular physical exercise				
Yes	330 (78.0)	93 (22.0)	1	1
No	127 (67.6)	61 (32.4)	1.58 (1.40, 5.48)	1.46 (1.29, 5.10)
Prolonged standing during session				
Yes	363 (77.7)	104 (22.3)	2.85 (1.34, 7.46)	4.78 (3.75, 9.32)
No	92 (63.9)	52 (36.1)	1	1
Length of breaking time between session				
Less than 15 minutes	202 (78.9)	54 (21.1)	2.35 (1.47, 3.74)	2.88 (0.68, 7.66)
Up to 1 hour	177 (77.6)	51 (22.4)	2.18 (1.35, 3.50)	1.61 (0.59, 4.35)
More than 1 hour	66 (64.1)	37 (35.9)	1	1
Have asthma				
Yes	84 (83.2)	17 (16.8)	1.81 (1.04, 3.16)	0.95 (0.28, 3.25)
No	373 (73.1)	137 (26.9)	1	1
Previous back pain injury				
Yes	144 (87.8)	54 (12.2)	2.36 (1.23, 4.53)	2.11 (0.36, 4.88)
No	226 (54.7)	187 (45.3)	1	1

∗ indicates those respondents who are divorced/widowed.

## Data Availability

The data used to support the findings of this study are available from the corresponding author upon request.

## Abbreviations

AOR: Adjusted odds ratio
BMI: Body mass index
CI: Confidence interval
COR: Crude odds ratio
LBP: Low back pain
SPSS: Statistical Package for Social Sciences
WHO: World Health Organization.
